# Mechanical Properties and Durability Performance of Concrete Containing Calcium Carbide Residue and Nano Silica

**DOI:** 10.3390/ma14226960

**Published:** 2021-11-17

**Authors:** Musa Adamu, Yasser E. Ibrahim, Mohamed E. Al-Atroush, Hani Alanazi

**Affiliations:** 1 Engineering Management Department, College of Engineering, Prince Sultan University, Riyadh 11586, Saudi Arabia; ymansour@psu.edu.sa (Y.E.I.); mezzat@psu.edu.sa (M.E.A.-A.); 2 Department of Civil Engineering, Bayero University, PMB 3011, Kano 700006, Nigeria; 3 Department of Civil and Environmental Engineering, College of Engineering, Majmaah University, Al-Majmaah 11952, Saudi Arabia; hm.alanazi@mu.edu.sa

**Keywords:** calcium carbide residue, nano silica, pozzolanic reaction, calcium hydroxide, compressive strength

## Abstract

Calcium carbide residue (CCR) is the end-product of production of acetylene gas for the applications such as welding, lighting, ripening of fruits, and cutting of metals. Due to its high pH value, disposing of CCR as a landfill increases the alkalinity of the environment. Therefore, due to its high calcium content, CCR is mostly blended with other pozzolanic materials, together with activators as binders in the cement matrix. In this study, cement was partially substituted using CCR at 0%, 7.5%, 15%, 22.5% and 30% by weight replacement, and nano silica (NS) was utilized as an additive by weight of binder materials at 0%, 1%, 2%, 3% and 4%. The properties considered were the slump, the compressive strength, the flexural strength, the splitting tensile strength, the modulus of elasticity, and the water absorption capacity. The microstructural properties of the concrete were also examined through FESEM and XRD analysis. The results showed that both CCR and NS increase the concrete’s water demand, hence reducing its workability. Mixes containing up to 15% CCR only showed improved mechanical properties. The combination of CCR and NS significantly improved the mechanical properties and decreased the concrete’s water absorption through improved pozzolanic reactivity as verified by the FESEM and XRD results. Furthermore, the microstructure of the concrete was explored, and the pores were refined by the pozzolanic reaction products. The optimum mix combination was obtained by replacing 15% cement using CCR and the addition of 2% NS by weight of cementitious materials. Therefore, using a hybrid of CCR and NS in concrete will result in reduction of cement utilization in concrete, leading to improved environmental sustainability and economy.

## 1. Introduction

Infrastructural development is significantly improving globally as most countries are moving towards urbanization. This leads to a rapid surge in the use of concrete, being the most widely utilized construction material. This, therefore, leads to the higher demand and utilization of cement as it is the main constituent material for concrete and mortar production. However, cement is the most environmentally unfriendly and expensive constituent material in concrete. The world is now focusing more on environmental and natural sustainable materials to address the rapidly increasing infrastructural demand. This, therefore, gives researchers a challenge in improving and achieving sustainability in the building and construction industries. It was projected that global demand for concrete will reach 18 billion tons annually by 2050 [1]. This will boost cement production and consequently increase global warming and environmental pollution through CO_2_ emission to the environment from the cement industries. According to Andrew [2] and Majhi et al. [3], about 5% of the total global CO_2_ emission comes from the cement industry, where approximately 1.25 tons of CO_2_ is generated from producing one ton of cement. In other reports by Andrew [2] and Le Quéré et al. [4], cement industries contributed about 8% of the overall global greenhouse CO_2_ emission, where 90% of the emission from industries comes from the cement factories [5]. Therefore, a series of research has been conducted trying to reduce the dependency of cement for concrete production. Several materials were used as a supplement to cement in concrete. Some of the materials have been promising towards improving the properties of concrete when replacing cement. Several agro and Industrial by-products have been used as supplementary cementitious materials (SCM). Some of the SCMs used in concrete with promising results include slag, silica fume, fly ash, natural Pozzolan, NS, rice husk ash, and CCR. The SCMs, due to their pozzolanicity, have the ability to react with the calcium hydroxide generated during cement hydration to produce more products of hydration responsible for strength development and improving the properties of the ITZ between cement paste and aggregate. Among the SCMs, nanomaterials, such as NS due it its high surface area, have higher pozzolanic reactivity, thereby fastening cement hydration and improving the properties of concrete better [6]

CCR is the end product of production of acetylene gas used for many applications such as welding, lighting, fruits ripening, and metals cutting. The production of CCR involves a reaction occurring chemically between water and calcium carbide (CaC_2_) to generate C_2_H_2_ (acetylene gas) and Ca(OH)_2_ (calcium hydroxide), as presented in Equation (1) [7,8,9].

(1) $${\mathrm{CaC}}_{2}+2H_{2}O--\to C_{2}H_{2}+\mathrm{Ca}{\left(\mathrm{OH}\right)}_{2}$$

Due to its high alkalinity, utilization of CCR for other applications seems unsuitable and is typically disposed of as landfills, mostly in slurry form. Due to its high pH value, CCR raises the alkalinity of the disposal area. It also causes health threats as it flies as dust into the environment [7,10,11]. Therefore, there is a need for proper utilization of CCR, and one of the effective ways is by applying it to cementitious materials such as concrete, mortar, geopolymer concrete, or soil stabilization. CCR is utilized as a promoter of calcium for improving strength development, where it produces excess Ca(OH)_2_ as a hydration product. The Ca(OH)_2_ in the presence of SiO_2_ and Al_2_O_3_ from cement or other pozzolanic materials generates excess compounds (C-S-H and C-A-S-H), the main products for strength improvement in cementitious matrix [12,13]. Therefore, CCR is mostly used in combination with other pozzolanic materials in cementitious composites.

Khongpermgoson et al. [14] developed a new binder material using ground bottom ash (GBA) and CCR at 70:30 ratio and used in combination with 10% cement in concrete. The strength of the new binder was less than the conventional concrete but can be used for structural applications. Furthermore, they reported a decrease in permeability of the concrete containing GBA plus CCR plus 10% OPC compared to the 100% OPC concrete. Karthiga et al. [15] studied the combined effects of CCR and slag as a substitute to cement in concrete by replacing cement using CCR (0%, 5%, 10%, 15% and 20% by weight), and slag (0%, 10%. 20% and 30% by weight). The compressive strength of the concrete increased with the incorporation of CCR and slag, where the optimum combination of the two materials was 35% (15% CCR with 20% slag), where the compressive strength increases by 27%, 13% and 15%, respectively, after 14, 28 and 48 days, respectively. Adamu et al. [16], investigated the effect of CCR and RHA as cement replacements in pervious concrete. They used replacement levels of 0%, 5%, 10%, 15%, and 20% by weight cement for both CCR and RHA. The consistency of the concrete decreases with an increase in both RHA and CCR. Furthermore, when only RHA was used as SCM, the mechanical strengths decreased. However, the addition of CCR to the concrete containing RHA significantly improves the strengths. However, the optimum dosage of CCR and RHA was 15% and 10%, respectively. In a similar study, Adamu et al. [17] also investigated the effect of CCR and RHA as SCM in pervious concrete, where they used replacement levels of 0%, 5%, 10%, 15% and 20% by weight. There was an enhancement in the strength and decrease in water absorption and permeability with the increase in both CCR and RHA. Based on mix optimization, they concluded that a combination of 0% RHA with 10% CCR yielded the best results in terms of durability performance.

Based on the existing literature, different pozzolanic materials have been used in combination with CCR to enhance the reaction between the Ca(OH)_2_ generated by the CCR during hydration with the SiO_2_ and Al_2_O_3_ from the pozzolanic material to produce excess hydration products for strength development. However, based on the findings from the literature, only a small replacement of CCR with other Pozzolanic material yielded good results. Therefore, Nano silica due to its very high SiO_2_ content, finer size and high reactivity [18], if used is expected to significantly improve the strength development in concrete or mortar containing CCR as SCM.

Nanotechnology is continuously becoming more acceptable in the construction and building industries. It is used in the form of nanomaterials in concrete and mortar to enhance the durability and mechanical performance of the concrete. It also improves environmental and material sustainability through the decrease in the use of cement in concrete and achieving higher strength at early ages with less cement content. Furthermore, nanomaterials significantly activate and enhance the pozzolanic reactivity of SCMs such as fly ash, slag, and RHA in concrete [19,20,21,22]. There are different nanomaterials used as additive and SCM in concrete to improve its properties at macro and nano levels, with nano-silica being the most used nanomaterial [20]. Additionally, nano silica has a very good filler ability and acting nucleation site effects, leading to pore reduction, microstructural and interfacial transition zone refinements [23,24]. Research by AlKhatib et al. [25] showed that 5% NS as additive by weight of binder activated the pozzolanic reactivity of electric arc furnace dust (EAFD) and cement kiln dust (CKD) when used as SCM in concrete and enhanced the strengths. Adamu et al. [18] recorded enhancement in the strengths of roller-compacted concrete made with HVFA and the addition of NS. Based on mix optimization carried out, they found a combination of 54% HVFA and 1.22% NS was the optimum combination. Similarly, According to Gunasekara et al. [26], the addition of 3% NS increases the strength of concrete containing 65% HVFA as SCM increased its strength by 50% and 10.3% at 7 and 28 days, respectively, and for concrete containing 80% HVFA by 98.6% and 35.9% at 7 and 28 days, respectively. They concluded that NS activated the pozzolanic reactivity of fly ash by serving as a nucleation site effect and increasing the early reaction involving C_3_A and C_4_AF phases, and consuming Ca(OH)_2_ to densify the concrete microstructure. Murthi et al. [27] reported improvement in the setting time and strength development at early ages for high-performance concrete with bagasse ash as SCM and the addition of NS by weight of binder. Shahrul et al. [28] investigated the effect of NS addition on the mechanical properties of rubberized concrete. They reported enhancement in the mechanical strengths and elasticity, decreased shrinkage and porosity, improvement in the ITZ with incorporation of up to 2.5% NS. They attributed this improvement to the high pozzolanic reaction of NS. According to Adamu et al. [29] and Huang et al. [30], NS as an additive resulted in reduction in the water absorption, porosity, and abrasion resistance of HVFA concrete. They concluded that NS refined the pores and densified the concrete microstructure.

Based on the critical review of previous research work carried, very few or no available literature that studied the effect of partial replacement of cement with CCR and NS as additive in concrete. Therefore, there is a need to study the effect of the hybrid of CCR and NS as cementitious materials in concrete. This is due to the fact that according to the literature, both CCR and NS when used with other Pozzolanic materials in concrete, significantly improved concrete’s properties. NS activates the pozzolanic reactivity of the SCMs in concrete, while CCR produces excess Ca(OH)_2_ which is utilized for pozzolanic reaction in concrete. Hence, in this research work, the effect of CCR as a partial substitute to cement and NS as addition by binder weight on the mechanical properties of concrete was investigated.

## 2. Materials and Methods

### 2.1. Materials

**Figure 1:**
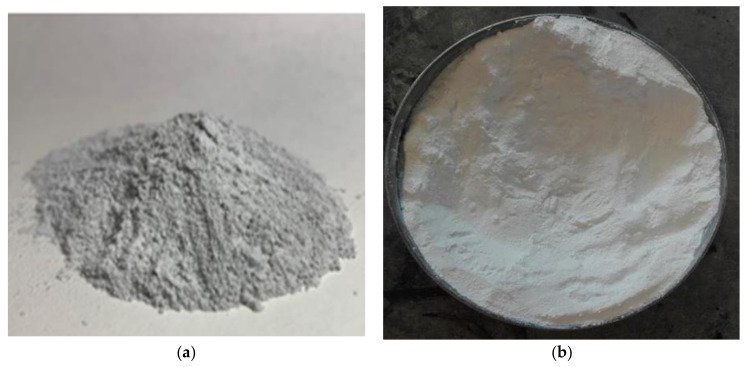
CCR and NS. (**a**) Calcium carbide residue. (**b**) Nano silica.

Ordinary Portland Cement Type 1, which satisfied the standard specifications of ASTM C150 [31] and which is readily available was utilized. Table 1 showed the properties of the cement. The cement was stored in a moisture-free and airtight container before usage to avoid lumping and hardening. The calcium carbide residue (CCR) was obtained from landfill disposal generated by a commercial welding workshop in Kano State, Nigeria. The CCR was readily available in abundance. The CCR was first air dried for up to four days and then oven-dried for another 24 h at 110 ± 5 °C to ensure it was totally free from moisture and completely dried. After drying, the CCR was grounded continuously in a grinding machine; only about 15% of its total weight was retained on No. 325 (45 µm) sieve. This method was adopted by Khongpermgoson et al. [14], and Namarak et al. [32]. The final processed CCR powder is shown in Figure 1a. The properties of the CCR were also presented in Table 1. Nano silica (NS) was utilized as an additive by weight of cementitious materials. The NS is hydrophobic in nature, in powdered form, and whitish in color with particle sizes ranging from 10–25 mm as shown in Figure 1b. The properties of the NS as obtained from the supplier were also presented in Table 1.

**Figure 2:**
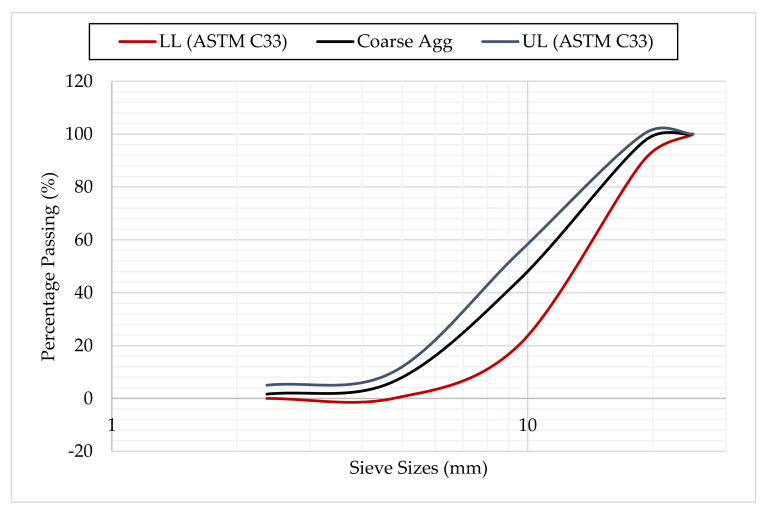
Particle Size Distribution of Coarse Aggregate.

**Figure 3:**
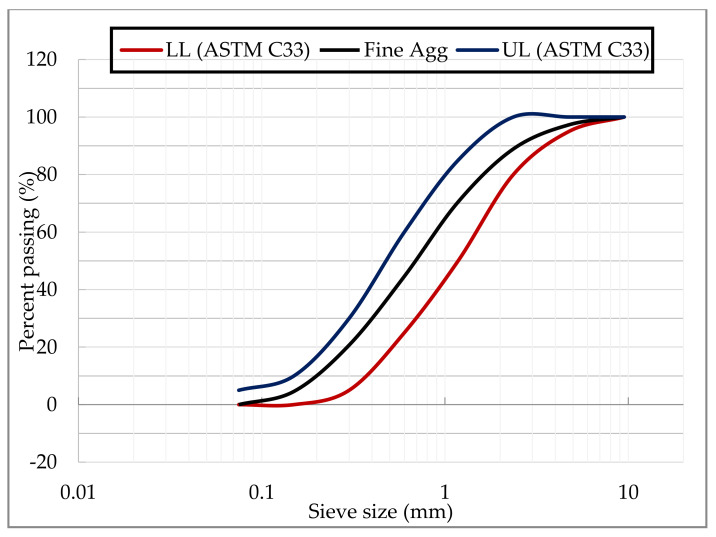
Particle Size Distribution of Fine Aggregate.

The coarse aggregate used was crushed granite having a maximum size of 19 mm which was free from dust and impurities. The properties of the coarse aggregate as obtained reference to ASTM C127 [33] were presented in Table 2. ASTM C136 [34] was used to obtain the particle size distribution (PSD) of the coarse aggregate as presented in Figure 2, where it falls within the recommended limits of ASTM C33 [35]. The fine aggregate used was a natural river sand, which was free from impurities. The PSD of the fine aggregate as determined with reference to ASTM C136 [34] is presented in Figure 3, where it falls within the recommended limits of ASTM C33 [35].

A Superplasticizer of polycarboxylate-based group which satisfied the BS EN 934-2 [36] specifications was used to decrease the quantity of mixing water needed and lower the ratio of the water-to-cementitious materials and enhance the strength. The superplasticizer has a density of 1.11 kg/l and pH value of 6.2 of 6.2 ± 0.5. The quantity of superplasticizer was 1% by weight of binder, and the amount of mixing water was lowered by 10%.

### 2.2. Mix Proportioning

The control mix was designed using the guidelines outlined in ACI 211.1R [37], for a target compressive strength of 30 MPa at 28 days. The absolute volume procedure for mix design was adopted. A water to cement ratio of 0.41 was maintained constant for all the mixes. The quantity of superplasticizer was 1% by binder materials weight and was used throughout the mixes to reduce the mixing water demand to achieve the required consistency.

To investigate the combined effects of calcium carbide residue (CCR) and nano silica (NS) on the properties of the concrete, twenty-five mixes were developed as shown in Table 3 using different variations of CCR and NS. The CCR was used as a substitute of cement at 0%, 7.5%, 15%, 22.5% and 30% by weight. NS addition was done at 0%, 1%, 2%, 3% and 4% by binder weight. All the mixes were labeled based on the amount of CCR and NS in it. For instance, M0C0N is the control mix having 0% CCR and 0% NS, mix M15C3N is a mix with 15% CCR and 3% NS, while mix M30C2N is a mix with 30% CCR and 2% NS.

### 2.3. Casting of Specimen

Prior to casting the concrete, the aggregates were brought to saturated surface dry conditions to prevent absorption of the mixing water, which can affect the consistency of the mixes and decrease strength. Before mixing, both cement and CCR were ensured to be fully dried and free from lumps and agglomerate. The mixing and curing were done based on the guidelines in ASTM C192/C192M [38] using a pan mixer type. Each of the constituent materials was weighed and batched. The mixing water was divided into two parts, the first part was mixed with the superplasticizer and nano silica (NS) and sonicated in an ultrasonicator for about 30 min to avoid agglomeration of the NS in the mix. The cement, CCR, and fine aggregate were firstly poured to the mixer and mixed for about 1 min, followed by coarse aggregate and of the half of the mixing water containing NS and superplasticizer was added and further mixed for about 3 min. As the mixing was taking place, the other part of the water was slowly added. The mixing was further continued for about 3 min until a homogenous and cohesive mix was achieved. Immediately after mixing, the slump was measured; then the fresh concrete was casted into the recommended molds. The fresh concrete in the molds were kept in the laboratory for at least 24 h, after which it was hardened before demolding. The hardened concrete samples were then stored in a curing tank containing clean water for the recommended days of curing before testing.

### 2.4. Test Methods

The workability of the fresh concrete was determined immediately after mixing using the slump test method outlined in ASTM C143/C143M [39].

The compressive strength test was conducted on each mix after 3, 7, and 28 days curing period using 100 mm cubes with reference to the methods outlined in BS EN 12390-3 [40]. A 2000 kN Universal Testing Machine (UTM) was utilized for the testing. Three samples were produced and tested for each curing period for each mix, and the average result was recorded.

The splitting tensile strength test was carried out based on specifications outlined in BS EN 12390-6 [41]. Cylindrical samples having dimensions 200 mm by 100 mm (height by diameter) were produced and tested after 7- and 28-day curing. A 2000 kN capacity UTM was also used for the splitting tensile strength. Furthermore, triplicate samples were prepared and tested for each of the mixes at each curing period.

For the flexural strength test, beams having dimensions of 100 mm × 100 mm × 500 mm were prepared and tested in accordance with the procedure outlined in ASTM C293/C293M [42], i.e., beam with center point load method. The samples were tested for flexural strength after a 7- and 28-day curing period in triplicates, and the average value recorded.

The methods outlined in ASTM C469 [43] were used to evaluate the modulus of elasticity for the concrete mixes after 28 days curing period. Cylindrical-shaped specimens 300 mm height by 150 mm diameter were produced in triplicate for each mix and used to measure the modulus of elasticity. Equation (2) was then used to obtain the modulus of elasticity after testing.

(2) $$E_{C}=\frac{\left(\sigma_{C}-\sigma_{1}\right)}{\left(\varepsilon_{2}-0.00005\right)}$$

where *E_c_* represents the modulus of elasticity; *σ_c_* represents the stress corresponding to 40% of maximum compressive force; *σ*_1_ represents the stress equivalent to the longitudinal strain of 0.00005, *ε*_2_ represents the longitudinal strain equivalent to *σ*_2_.

**Figure 4:**
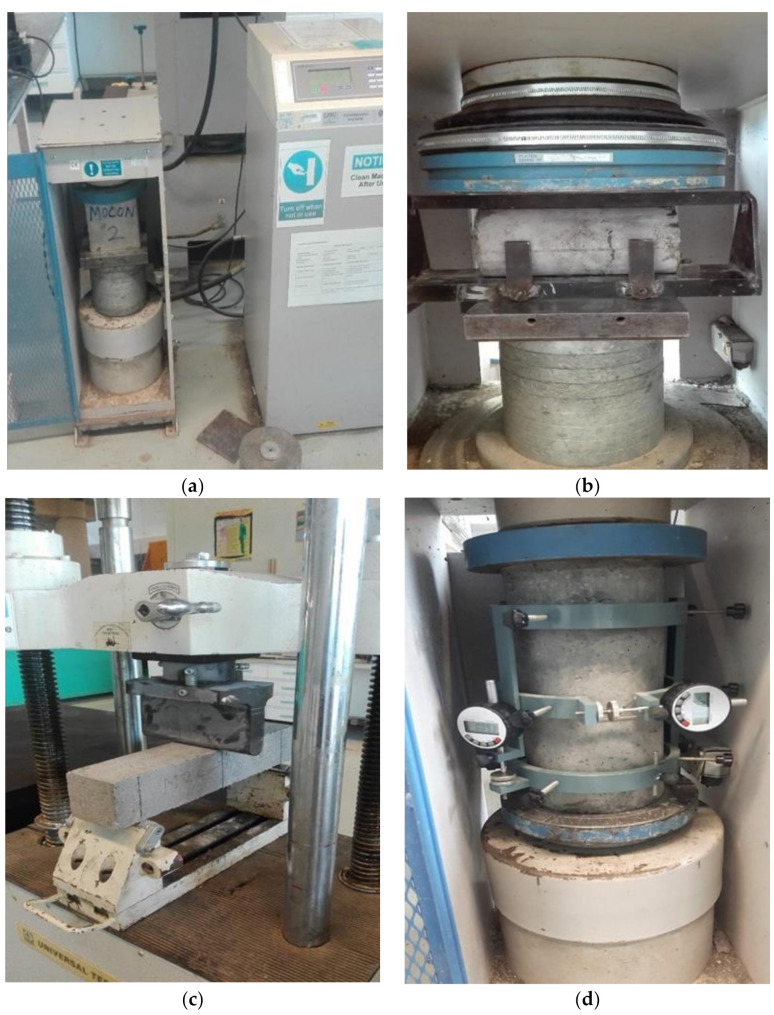
Experimental Setup. (**a**) Compressive Strength. (**b**) Splitting Tensile Strength. (**c**) Flexural Strength. (**d**) Modulus of Elasticity.

The water absorption test was conducted on all the mixes in accordance with the specifications of ASTM C642 [44] using 100 mm cube samples. For each of the mixes, three samples were produced and cured for 28 days prior to testing. The experimental set up for some selected tests are presented in Figure 4.

**Figure 5:**
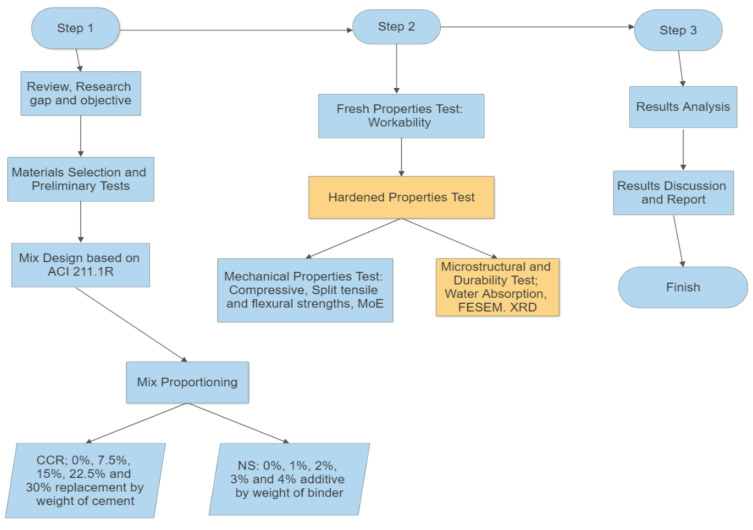
Flow Chart Methodology.

Field emission scanning electron microscope (FESEM) test was conducted on some selected mixes. Small fractures were extracted from the samples after 28 days of curing and allowed to dry completely, carrying out the FESEM test. Small pieces of the concrete were obtained after conducting the 28 days compressive strength testing. The concrete piece was cleaned and dried, and then coated with a thin gold film before placing it in the FESEM machine. During the test, morphology of the concrete in the form of high-resolution pictures for up to 10,000 magnifications were obtained. The FESM results were used to examine the effect of CCR and NS on the microstructure of the concrete, such as densification, interfacial transition zone, and traces of hydration products in the concrete matrix and pores. For the XRD analysis, few mixes were selected to study the effect of CCR and NS on the hydration products of the concrete. The test was carried out after a 28 day curing period, where the paste was extracted from the compressive strength test samples. The paste was then cleaned, dried, and grinded to a very fine powder. The XRD analysis was then conducted using an X’Pert3 MRD Materials Research X-ray Diffraction System. The schematic flow chart of the whole research work is presented in Figure 5.

## 3. Results and Discussions

### 3.1. Workability

**Figure 6:**
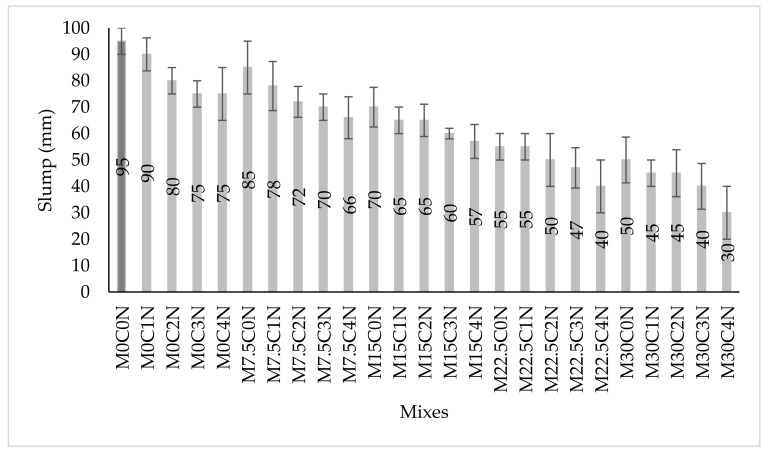
Slump Test Results.

The workability of the concrete containing calcium carbide residue (CCR) as a partial substitute to cement and nano silica (NS) as addition by weight of binder was measured using slump test, and the result presented in Figure 6. The slump values of the concrete have been decreased with the increase in partial substitution of cement using CCR. There was a decrease of 10.5%, 26.3%, 42.1%, and 47.4% for mixes M7.5C0N, M15C0N, M22.5C0N, and M30C0N, respectively, when compared to the control (M0C0N). The decrease in the slump due to CCR is due to higher surface area and loss of ignition value of the CCR, thereby absorbing much water when mixing, hence increasing water demand to obtain consistent mix thus decreasing slump [45,46]. Mokuolu et al. [47] also reported decreased workability with the replacement of cement using CCR, even though the slump of the concrete with up to 50% CCR was within the design range of 10–50 mm. The slump of the concrete further decreases with the addition of NS. As seen in Figure 6, mixes having higher CCR and NS had more reduction in slump compared to mix M0C0N. The reduction in the slump due to NS addition was mainly caused by the larger surface area of the NS resulting from its very fine sizes in comparison to both cement and CCR. Therefore, NS absorbs more water compared to both cement and CCR during mixing and increases the internal friction between the particles (both paste-to-paste and paste-to-aggregate). This leads to a decrease in the consistency of the mix and hence a reduction in the slump [18,48].

### 3.2. Compressive Strength

**Figure 7:**
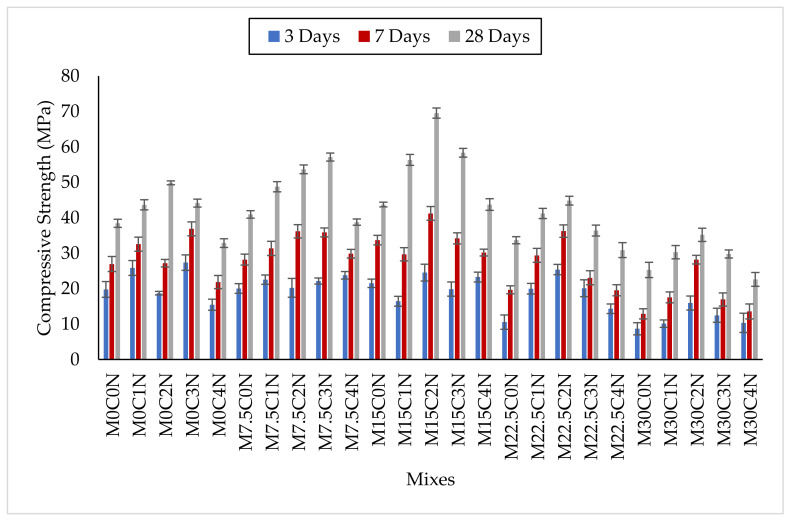
Compressive Strength Results.

The results of compressive strength for all the concrete mixes containing different percentages of CCR as supplementary cementitious material and NS by binder weight is presented in Figure 7. For each mix, the compressive strength increased with age of curing due to continuous hydration. The compressive strength of the concrete increased with partial replacement of up to 15% cement using CCR without any NS addition. For 7.5% replacement, the compressive strength of mix M7.5C0N was higher by 1.4%, 4.6%, and 6.5% at 3, 7, and 28 days, respectively, when compared to the control (M0C0N). Similarly, the compressive strength of M15C0N improved by 8.8%, 25.1%, and 13.8% at 3, 7, and 28 days, respectively, compared to M0C0N. The improvement in compressive strength is due to CCR contains higher amount of calcium oxide (CaO), thereby producing more alkaline compound Ca(OH)_2_, which further reacts with the little SiO_2_, and Al_2_O_3_ from the cement to form more hydration products such as C-S-H and C-A-S-H which are the major contributors for strength development and pore filling in concrete [16,49]. Karthiga et al. [15] also reported an increase in compressive strength by 3%, 4.42% and 13% when cement was replaced 5%, 10% and 15%, respectively, using CCR. Similar findings have been reported by Mokuolu et al. [47], where they also observed improvement in compressive strength with the replacement of up to 20% cement using CCR. Krammart and Tangtermsirikul [50] also found that the strength of mortar made with up to 10% CCR as cement replacement demonstrated similar strength as the conventional mortar. Amnadnua et al. [51], also reported improvement in compressive strength of concrete at later ages from 28 days when up to 20% CCR was used. On the other hand, when a higher amount of CCR above 15% was used to replace cement, there was decline in the compressive strength at all ages of curing. The strength of M22.5C0N was lower compared to M0C0N by about 46.7%, 27%, and 12.4% at 3, 7 and 28 days, respectively. In addition, the strength of M30C0N was less by 56.1%, 52.2% and 34.2% at 3, 7 and 28 days, respectively, when compared to M0C0N. The decrease in strength when higher CCR was added was more noticeable at earlier ages, and this could be due to the higher amount of Ca(OH)_2_ produced at the early age of the hydration reaction, which hinders strength development. Li and Yi [52] also reported similar findings. With increased curing and hydration, the amount of Ca(OH)_2_ kept diminishing; thereby the reduction in strength also lessens.

NS addition to the concrete mixes containing CCR as SCM significantly improved the compressive strength all ages. Even for the mixes containing up to 22.5% CCR, NS improved the compressive strength to be greater than the control mix. The addition of 1% and 2% NS to mixes containing 22.5% CCR such as M22.5C1N and M2.5C2N improved their strength. At 3, 7, and 28 days, the strengths of M22.5C1N were higher than those of the control by 1.1%, 9.1%, and 7.2%, respectively. While the strengths of M2.5C2N were higher than for the control mix by 28.4%, 34.5%, and 16.6%, respectively. The reasons for the improvement in strength with the addition of NS was due to the higher SiO_2_ content and high reactivity of NS, thereby reacting with the excess Ca(OH)_2_ produced by the CCR during hydration reaction to make more hydration products such as C-S-H gels which densified the pores in the hardened microstructure and improves strength develop in the concrete. Furthermore, NS itself, due to its very fine particle sizes, had pore filling ability thereby densifying the microstructure of the concrete and increasing strength [20,53,54]. On the contrary, when a higher dosage of NS was incorporated to the concrete mixes containing CCR, there was decline in compressive strength. This was more pronounced in the mixes containing higher CCR contents. For instance, the addition of 3% and 4% NS to the concrete mix containing 30% CCR further decreases its strength as seen in Figure 7. This was caused by the agglomeration of NS due to less water available for mixing. As higher CCR was used, the workability decreased due to the higher loss of ignition, and with NS also requiring more water for mixing due to its larger surface area, this leads to poor consistency and agglomeration in the mix. This caused improper dispersion of the paste in the concrete matrix, resulting in a porosity and possibly honeycomb in the hardened matrix, and consequently decline in the strength. Additionally, the absorption of the mixing water by the higher contents of CCR and NS also reduced the hydration and pozzolanic reaction in the concrete, hence diminishing the production of C-S-H gels and consumption of Ca(OH)_2_, thereby leading to lower strength development. Nazari and Riahi [55] also reported similar observations when they used NS in concrete containing slag, and Adamu et al. [18] for NS in fly ash concrete.

### 3.3. Splitting Tensile Strength

**Figure 8:**
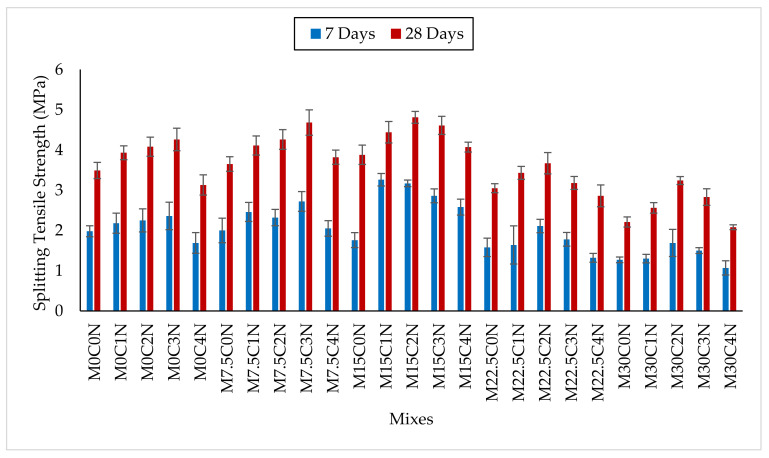
Splitting Tensile Strength Results.

Figure 8 presents the results for the splitting tensile strength, where it increased with age due to the continual hydration of the cementitious materials. Substitution of up to 15% cement using CCR enhanced the splitting tensile strength. The splitting tensile strengths of M7.5C0N were more than for the control (M0C0N) by about 5.6% and 4.6% at 7 and 28 days, respectively. Similarly, for mix M15C0N, the splitting tensile strength values were higher by 6.6% and 11.2% at 7 and 28 days, respectively, when compared to M0C0N. The high CaO in CCR produced much Ca(OH)_2,_ which reacts with the SiO_2_ and Al_2_O_3_ in cement to generate additional C-S-H. The C-S-H filled up the pores in the concrete matrix and refined it, densified the ITZ between the cement paste and aggregate, and hence enhanced the compressive strength and hence improved tensile strength. Abdulmatin et al. [46] also reported that the tensile strength of concrete made with binder containing fly ash and CCR is dependent and directly proportional to its compressive strength. Adamu et al. [16] also reported an increase in splitting tensile strength of pervious concrete where they found an improvement by up to 63% with replacement of 10% cement using CCR in pervious concrete containing rice husk ash. In contrast, a higher content of CCR as SCM in the concrete resulted in reduction of tensile strength. As the cement content is further reduced, less elements that produce the hydration products are available, thereby leaving excess Ca(OH)_2_ as a by-product of hydration. This Ca(OH)_2_ leached out easily, therefore increasing porosity in the concrete’s microstructure and hence reducing the tensile strength.

NS addition to CCR concrete improved its splitting tensile strength at 7 and 28 days. However, this increment is for up to use of 15% CCR in the concrete. For example, considering mixes containing 15% CCR, the splitting tensile strength of mixes M15C1N, M15C2N, M15C3N, and M15C4N were higher by 21.3%, 36%, 30.8% and 3.3%, respectively, at 7 days and 11.1%, 24%, 14.4%, and 4.9%, respectively, at 28 days compared to mix M15C0N. The splitting tensile strength of mixes M22.5C1N, M22.5C2N, and M22.5C3N were higher by 12.1%, 6.6%, and 20.7%, respectively, at 7 days and 14.0%, 5.2% and 18.3% at 28 days, respectively, when compared to M0C0N. This improvement resulted from the high SiO_2_ (pozzolanicity) in NS reacting with the extra Ca(OH)_2_ generated by the CCR to make more C-S-H gels, which made the concrete microstructure denser by filling the pores, refining the ITZ between cement paste and aggregate, thereby improving bonding and consequently enhancing the splitting tensile strength. Furthermore, NS due to its pore filling ability from its finer size, also leads to densification of the microstructure and ITZ in the cement matrix, thereby increasing strengths.

**Figure 9:**
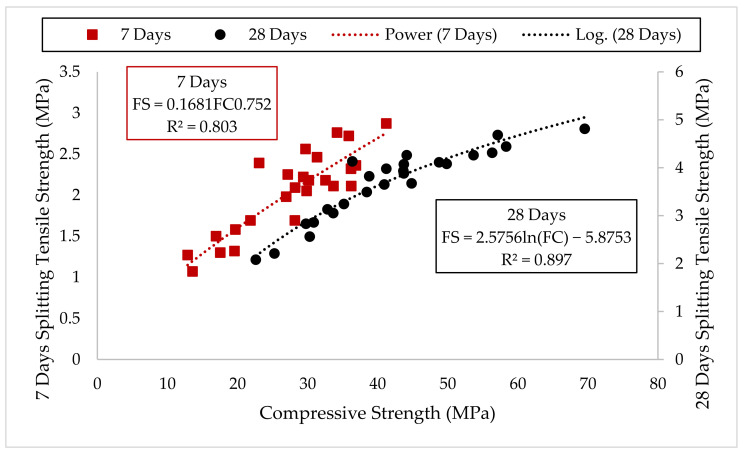
Relationship between splitting tensile strength and compressive strength.

The relationship between 7 and 28 days splitting tensile strength versus 7- and 28-days compressive strength of the concrete mixes containing CCR and NS was presented in Figure 9. A good degree of correlation existed between the two strengths at both 7 and 28 days, with a proportionality between them.

### 3.4. Flexural Strength

**Figure 10:**
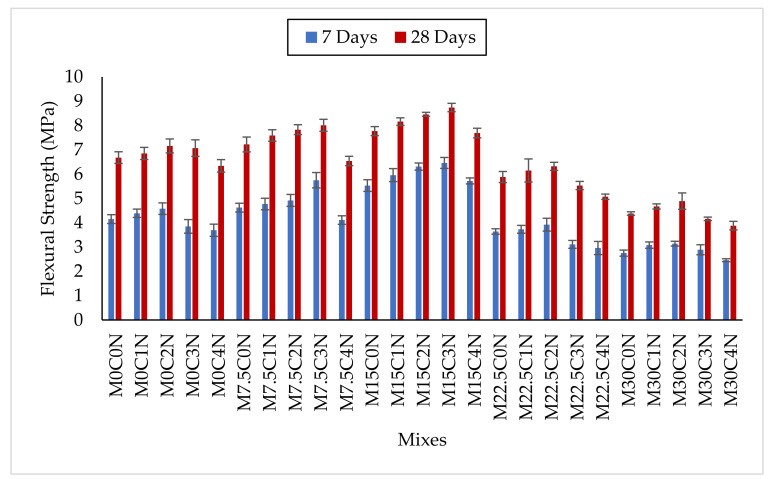
Flexural Strength Results.

Figure 10 presented the flexural strength results at 7 and 28 days, and it increased with age of curing due to the continuous hydration process. Substitution of up to 15% cement using CCR improved the bending resistance at 7 and 28 days. The flexural strengths of M7.5C0N and M15C0N were higher by about 11.3% and 26% at 7 days, respectively, and 8.1% and 16.3% at 28 days, respectively, when compared to control (M0C0N). The increase might be from the escalation in the generation of C-S-H gels during the hydration process, where CCR produced more Ca(OH)_2_, which reacts with some elements of the cement-like SiO_2_ and Al_2_O_3_ to generate extra C-S-H. The excess C-S-H further refined the microstructure and densified the paste-aggregate ITZ, which consequently leads to higher bending resistance. Adamu et al. [16] also found an improvement in the flexural strength of pervious concrete with partial replacement of cement using CCR, where up to 27% improvement was reported with addition of 10% CCR. For mixes containing 22.5% and 30% CCR without any NS addition, the flexural strength decreased compared to M0C0N, and this can be due to the excess Ca(OH)_2_ generated during hydration, which can be leached out easily, leaving pores in the hardened matrix. This will result in higher porosity in the matrix and consequently reduce the flexural strength.

NS addition further enhanced the flexural strength of the mixes containing any percentage of CCR. For instance, the flexural strength of M22.5C1N, M22.5C2N, and M22.5C3N were greater than M0C0N by 4.6%, 12.3%, and 20%, respectively, at 7 days and 8.2%, 14.7%, and 18.7%, respectively, at 28 days. The addition of up to 2% NS to mixes containing 30% CCR improved its strength. M30C1N had higher flexural strength than M30C0N by 12% and 6.6% at 7 and 28 days, respectively, while M30C2N had higher values compared to M30C0N by 14.2% and 11.6% at 7 and 28 days, respectively. The increase in flexural strength resulted from the densification and enhancement of the ITZ and improving adhesion between aggregate and paste which significantly increases the bending resistance. This is due to the excess hydration products generated from the pozzolanic reaction between SiO_2_ from NS and Ca(OH)_2_ from CCR [18]. However, mixes containing a higher content of CCR with higher NS, such as M22.5C4N and M30C4N showed a reduction in flexural strength, and this can be due to agglomeration of NS, and reduction inconsistency due to the higher water absorption of CCR and NS. This causes improper dispersion of the paste within the cement matrix, thereby creating pores or honeycombs and consequently bending failure with the application of load.

**Figure 11:**
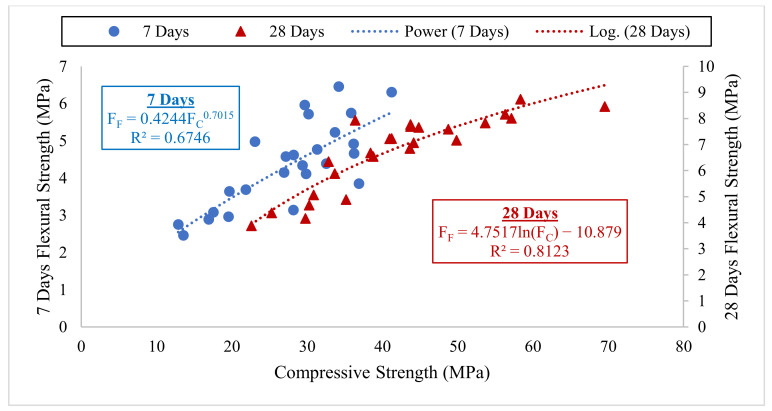
Relationship between Flexural strength and compressive strength.

Figure 11 showed the relationship between compressive and flexural strengths. A good degree of correlation existed between the 28 days strengths, while a fair correlation existed between the 7 days strengths, with a direct proportion between them.

### 3.5. Modulus of Elasticity

**Figure 12:**
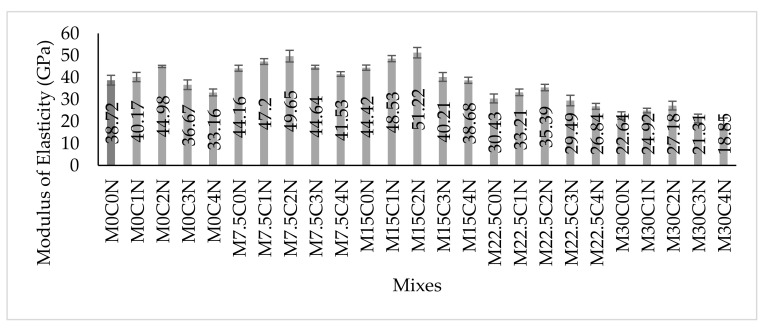
Modulus of Elasticity Results.

Figure 12 presented the results of the average modulus of elasticity (MoE) of the concrete. As it has already been established that the MoE is directly proportional and dependent on the compressive strength of concrete [1,56,57,58]. Substitution of up to 15% cement using CCR enhanced the MoE of the concrete without any NS addition. Compared to the control (M0C0N), the MoE of M7.5C0N, and M15C0N were greater by 11.5% and 14.7%, respectively. This increase was due to the increased reaction between the Ca(OH)_2_ from the CaO in CCR and some elements in cement-like SiO_2_ and Al_2_O_3_. This resulted in increasing in C-S-H contents, which densified the concrete microstructure and consequently increased the strength and stiffness, thereby enhancing the MoE of the mixes. However, higher CCR contents from 15% without any NS addition resulted in a decrease in MoE, as the MoE values of M22.5C0N and M30C0N were lower than for the control mix by about 21.4% and 41.5%, respectively. This decrease might be due to excess Ca(OH)_2,_ which could not react with the elements from cement and thereby leached out easily, hence creating pores in the concrete microstructure. These pores caused a decline in strength and stiffness of the concrete and hence decreased its MoE. Contrary to these findings, Amnadnua et al. [51] reported that CCR has not much effect on the MoE of concrete, and they attributed their reasoning to the fact that the strength of concrete is dependent on the aggregate strength and not the cement paste.

NS addition significantly improved the MoE of the concrete mixes containing CCR. Therefore, with NS as an additive, up to 22.5% CCR as SCM can be used to improve the stiffness and MoE of concrete. As shown in Figure 12 where the MoE of mixes M22.5C1N, M22.5C2N and M22.5C3N were more than for the control by about 11.6%, 17.2% and 3.1%, respectively. Furthermore, compared to mix M15C0N, the MoE of mixes M15C1N, M15C2N and M15C3N were higher by about 25.3%, 32.3% and 14.2%, respectively. The increase in MoE due to addition of NS might be due to the higher reaction between the NS (high SiO_2_) and CCR (high Ca(OH)_2_ from its high CaO), creating supplementary hydration products filling the porosity in the concrete and densified its microstructure thereby increasing strength and stiffness and consequently higher MoE [18,54]. Adamu et al. [18] also reported improvement in the MoE of HVFA concrete when NS was used as an additive, where the NS ignited the pozzolanic reaction in the cement paste and improved strengths.

**Figure 13:**
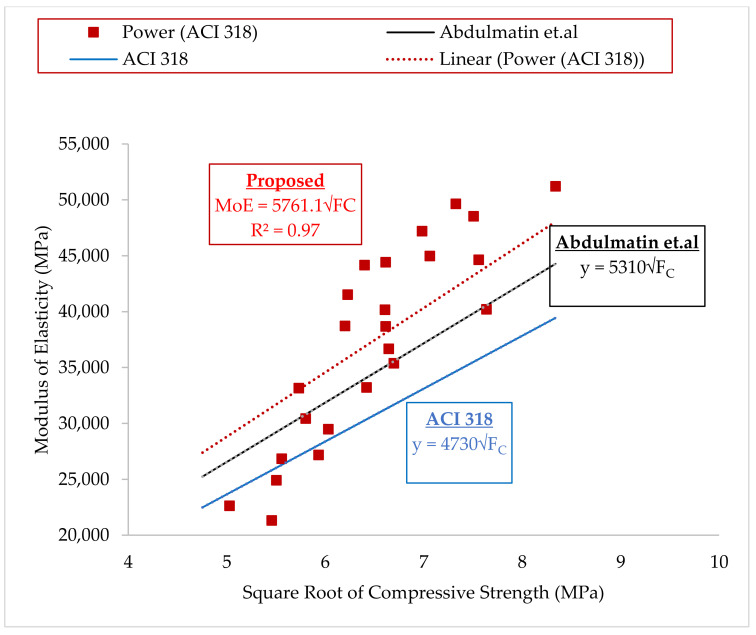
Relationship between Compressive Strength and Modulus of Elasticity.

The relationship between the compressive strength and MoE of the concrete is presented in Figure 13. Power model was proposed, with the model having a good degree of correlation (R^2^). The Power model as given in Eqn 3 can be compared to the models for normal concrete according to ACI 318 as given in Eqn 4 [59] and for high strength concrete according to ACI 363 as given in Eqn 5 [60], where it can be seen that the variation between the coefficient from the developed equation is higher than for ACI 318, as most of the compressive strength values fall within high strength concrete. Compared with Eqn 5, the coefficient for the proposed model was slightly higher and this might be because of the materials used in the concrete, which caused variations in the strength and elasticity. Comparing the developed relationship between the compressive strength and MoE with that developed by Abdulmatin et al. [46] for concrete containing bottom ash and CCR as seen in Figure 13, there is a slight variation between the coefficients and this might be due to the fact that for this study a combination of CCR and NS was used while for Abdulmatin et al. [46], a combination of CCR and fly ash was used. Based on this, it is expected that each of the concrete will exhibit different behaviors and different strengths.

(3) $$\mathrm{Proposed}\;\;\;\;\;\;\;\;\;\;\;\;\;\;\;\;\;\;\;\;\;\;\;E_{C}=5761\sqrt{F_{C}}$$

(4) $$\mathrm{ACI}\;318\;\;\;\;\;\;\;E_{C}=4730\sqrt{F_{C}}$$

(5) $$\mathrm{ACI}\;363\;\;\;\;\;\;\;\;\;\;\;\;\;\;\;\;\;\;\;\;\;E_{C}=5600\sqrt{F_{C}}$$

where *E_C_* is the Modulus of Elasticity in MPa, and *F_C_* is the compressive strength in MPa.

### 3.6. Water Absorption

**Figure 14:**
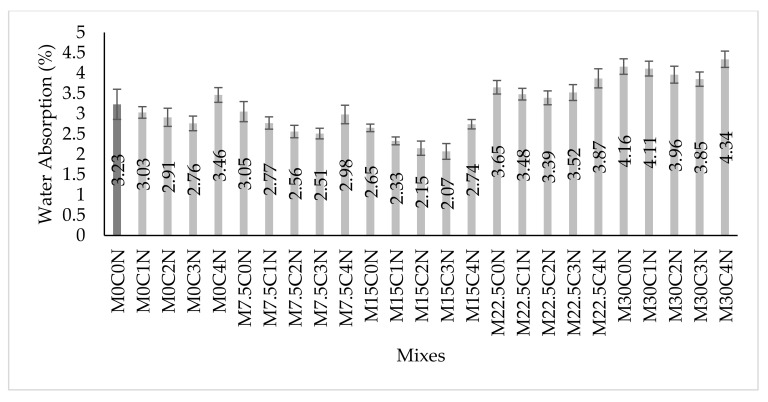
Water Absorption Results.

Figure 14 presented the water absorption of the concrete. Substitution of cement partially using CCR decreased the water absorption, where for mixes M7.5C0N and M15C0N their values were lower than the control mix by about 5.6% and 18%, respectively. This decrease might be attributed to the physical properties of the CCR, where due to its finer sizes having pore filling ability, it densified the concrete microstructure and refined the pores, thus decreasing the water ability. Another reason might be due to the chemical reaction of CCR, which, during hydration, reacts with the elements in cement to produce more hydration products which densifies the concrete microstructure, reduces porosity, and hence decreases the water absorption. However, for 22.5% and 30% CCR, the water absorption increases and this might be due to excess Ca(OH)_2_ in the cement matrix, which increases porosity and water absorption.

The addition of NS to mixes containing CCR further decreased their water absorption values. For instance, mixes containing 7.5% CCR, the water absorption values of M7.5C1N, M7.5C2N, M7.5C3N and M7.5C4N were lower by 9.2%, 16.1%, 17.3%, and 2.3%, respectively, in comparison to mix M7.5C0N. Additionally, in comparison to the control mix, the water absorption of M22.5C1N, M22.5C2N, M22.5C3N and M22.5C4N were lower by 4.6%, 13.6%, 22%, and 7.7%, respectively. The decreases in water absorption might be attributed to the physical properties of NS were due to its very fine sizes, it could fill the pores and densify the cement matrix, hence decreasing the water absorption. Another reason can be due to the chemical properties of the NS, where its high SiO_2_ reacts with the hydration products from CCR and cement, such as crystalline Ca(OH)_2_, producing excess amorphous C-S-H, filling the nanopores in the cement matrix, producing a more homogenized and densified microstructure, thereby decreasing water absorption [29,54].

**Figure 15:**
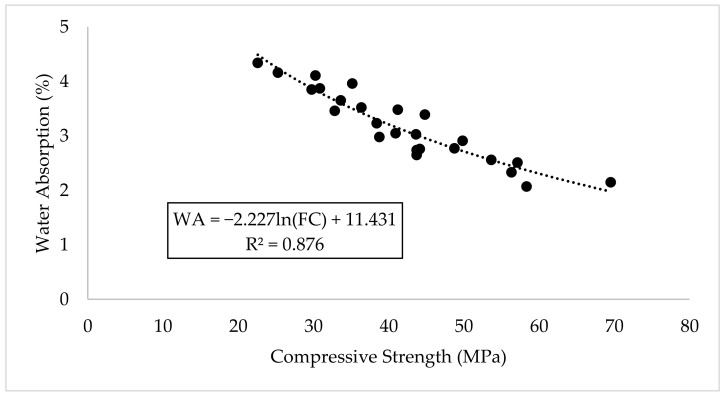
Relationship between Compressive Strength and Water Absorption.

Figure 15 showed the relationship between the compressive strength and water absorption of the concrete, where a high level of correlation existed between them. The higher the strength, the lower the water absorption and hence the denser the concrete microstructure.

### 3.7. Microstructural Properties (FESEM)

**Figure 16:**
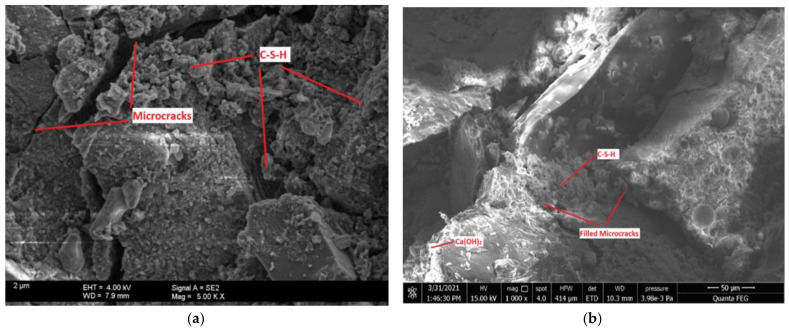

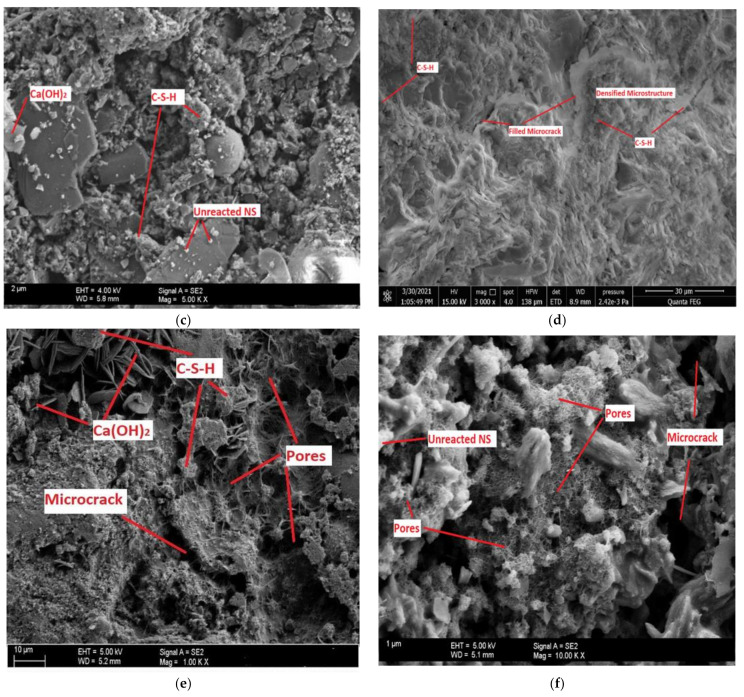
FESEM Microstructural Morphology. (**a**) Control Mix (M0C0N). (**b**) M7.5C2N. (**c**) M15C1N. (**d**) M15C2N. (**e**) M22.5C0N. (**f**) M30C4N.

The microstructural morphology of some selected representative mixes was studied using FESEM analysis, and the result is presented in Figure 16. The control mix has a densified microstructure due to the total hydration coming from cement; however, microcracks were observed at the surface of the hydration products as seen in Figure 16a. Wang et al. [61] made a similar observation. On the contrary, considering mix M7.5C2N, the microcracks were seen to be filled by the hydration products (C-S-H), and the microstructure was more densified compared to that of the control. This is due to the high pozzolanic reaction between the SiO_2_ and Ca(OH)_2_ produced during the hydration of the concrete mix containing CCR and NS. This reaction resulted in more C-S-H generation and consumption of Portlandite (Ca(OH)_2_), therefore a denser microstructure. Furthermore, it can be observed that the microstructure of M15C2N was the most densified, having the highest degree of pozzolanic reaction. Therefore, its microstructure was the most refined. This can be justified as mix M15C2N has the highest mechanical strengths and least water absorption value. From Figure 16e, plates of Ca(OH)_2_ (Portlandites) were found on the morphology of mix M22.5C0N. This is coming from the unreacted Ca(O)_2_ produced by the excess CCR during the hydration reaction which contains a high amount of CaO. In the absence of excess pozzolanic elements to react with the Ca(OH)_2_, the Ca(OH)_2_ easily leaked out, leaving pores inside the cement matrix as seen in the morphology of mix M22.5C0N. By comparing the morphology of mix M30C4N (Figure 16f), it can be observed its microstructure is the most porous, and this might be due to the fact that both CCR and NS absorb much water during mixing due to their high surface areas, in addition to the high loss of ignition of CCR. Therefore, during mixing the consistency of the mix was very low. Hence the paste was not easily dispersed within the concrete matrix. This causes pores in the hardened cement matrix. This can also be justified as mix M30C4N has the fewest mechanical strengths and highest porosity (water absorption) values.

### 3.8. Microstructural Properties (XRD Analysis)

**Figure 17:**
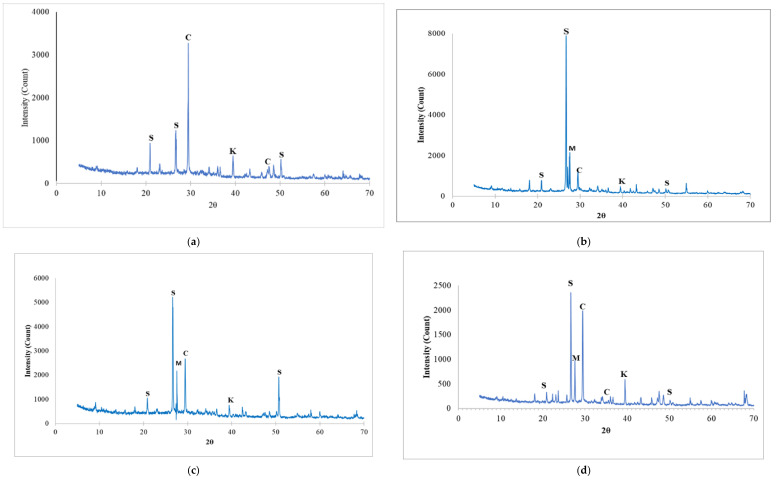

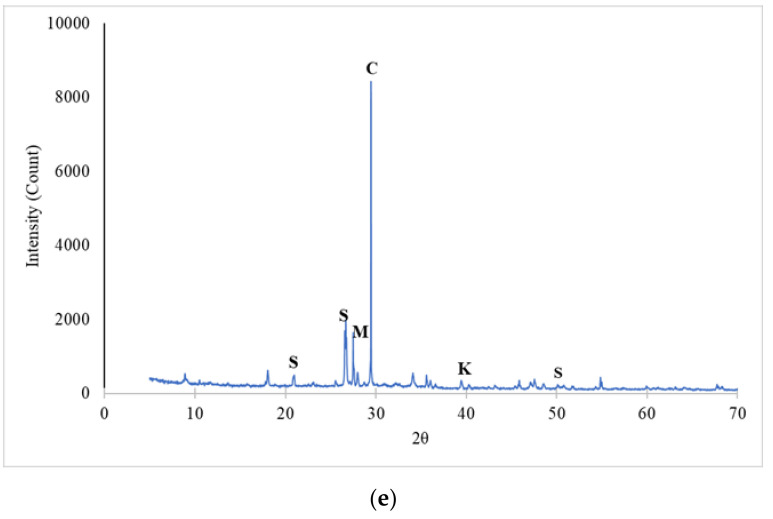
XRD Pattern of concrete mix containing CCR and NS. (**a**) M0C0N. (**b**) M0C3N. (**c**) M7.5C3N. (**d**) M15C2N. (**e**) M30C1N.

The X-ray Diffractometer (XRD) analysis was carried out to examine the effect of CCR and NS on the hydration reaction and products of the concrete. Representative mixes containing both CCR and NS were selected, and the results of the XRD analysis were presented in Figure 17. For the control mix, as seen in Figure 17a, the major hydration product elements were Ca(OH)_2_ (represented as C), SiO_2_ (represented as S), and CaCO_3_ (represented as K). The peak hydration product was the crystalline Ca(OH)_2_ occurring at about an angle (2-theta) of 28–30°. This is due to the CaO content in cement which reacts in the presence of water to give Portlandite as a by-product, with no supplementary pozzolanic material to consume it. Zhou et al. [62], also reported similar findings. With the addition of 3% NS to the concrete without any CCR addition, the peak of the Ca(OH)_2_ rapidly decreased, where quartz (SiO_2_) had the highest hump peak occurring between angles (2-theta) 26–28°, as seen in Figure 17b. This happens because of the pozzolanic reaction between SiO_2_ with the surplus Ca(OH)_2_ to produce additional C-S-H, amorphous in nature and hard to detect using XRD [63]. Figure 17d is the XRD for a mix containing 15% CCR and 2% NS, and it can be observed that two peaks of SiO_2_ and Ca(OH)_2_ were detected, having lower intensities compared to mixes containing high CCR with low NS, or low CCR with high NS. Therefore, there is a tendency that in mix M15C2N both SiO_2_ and Ca(OH)_2_ undergo a higher pozzolanic reaction and consume each other to generate more C-S-H gels. This can further be justified as mix M15C2N has the highest strengths values and lowest water absorption. Lastly, the XRD results for mix M30C1N (Figure 17e) have the highest intensity of Ca(OH)_2_, with the peak occurring at about 30–38° 2-theta. This is due to the fact that with more CCR, of about 30%, and less NS, of about 1%, the CCR, which contains a very high percentage of CaO, generated much excess Portlandites during hydration. Even though a pozzolanic reaction has taken place due to the presence of SiO_2_ from NS, the excess Ca(OH)_2_ was not adequately consumed. Therefore, during XRD analysis, a high-intensity peak of the crystalline Ca(OH)_2_ was detected. During the XRD analysis, other peaks of elements such as mullite (Al_6_Si_2_O_13_), represented as M, were detected, which might occur from reaction between Al_2_O_3_ from cement and SiO_2_ from NS. Additionally, CaCO_3_ (represented as K) was also detected in some mixes, which might also be from the reaction of cement elements with the CCR elements or from limestone used in producing cement.

### 3.9. Economic and Environmental Impact

Cost analysis was carried out for all the concrete mixes. Concrete mix proportions in Table 3 were used in combination with the current cost of each material, as presented in Table 4, to compute the cost of production of each mix in the laboratory only. The cost of each material was based on the most recent market prices. CCR was obtained free from commercial welders as they normally disposed of it as landfill. Water was also obtained free from the Laboratory. Table 5 presents the cost for producing each mix based on constituent materials. It can be seen that the cost of concrete production decreases with increment in replacement of cement using CCR. This is due to the fact that the CCR is a waste material and is inexpensive compared to cement. However, the addition of NS increases the cost of the concrete. Mixes with lower CCR contents show higher increment in cost due to addition of NS compared to mixes with higher CCR. This is attributed to the fact the higher content of the CCR significantly reduced the cost of the concrete even when NS was added.

One of the major disadvantages of using cement in cementitious composites is due to the high amount of greenhouse gas emissions to the environment. According to some studies for one ton of cement produced, an equivalent of 1 ton CO_2_ is emitted to the environment [64,65,66]. One of ways of reducing the CO_2_ emission from cement is by utilizing SCM in concrete. This will lead to reduction in the amount of cement produced and consequently lower CO_2_ emissions. In this study, the effects of CCR as partial substitute to cement and NS as additive on the CO_2_ emission of concrete were studied. The equivalent CO_2_ emissions from cradle to get for each of the mixes were calculated using Equation (6) in conjunction with the data in Table 3 and Table 4.

(6) $${\mathrm{CO}}_{2}=\sum_{i=1}^{n}(\chi_{i}\times {\mathrm{CO}}_{2\left(i\right)-\emptyset })$$

where *i* represents the mix number, *n* represents the total number of mixes, *χ_i_* represents the quantity of raw material *i* in kg/m^3^ and CO_2(*i*)−ø_ represents the CO_2_ emission of the raw material *i* in (CO_2_-kg/kg).

Table 5 presents the results of CO_2_ emissions from cradle to gate for all the concrete mixes. In comparison to the control mix (conventional concrete), the CO_2_ emissions of the concrete mixes decrease with increment in partial replacement of cement using CCR. This is due to the lower CO_2_ emission of the CCR compared to cement it replaced as shown in Table 4. Therefore, CCR is said to be a better environmentally sustainable material compared to cement. The addition of NS has a negligible effect on the CO_2_ emission of the concrete due to its very low CO_2_ emission as seen in Table 4. Therefore, NS addition very slightly increased the CO_2_ emission of the concrete mixes, which increases with the increment of NS addition.

## 4. Conclusions

The effect of partial substitution of cement using CCR and addition of NS by weight of binder on the mechanical properties of concrete was investigated. The following conclusions were drawn:

(1) The workability of concrete decreases with increment in partial substitution of cement using CCR, due to higher surface area and loss of ignition. NS addition to the concrete containing CCR further decreases the concrete’s workability, thereby increasing water demand during mixing due to its very fine sizes;
(2) The strengths and modulus of elasticity of concrete increased with replacement of up to 15% cement using CCR. On the contrary, higher replacement levels of cement with CCR above 15% resulted in a decrease in the listed properties;
(3) NS addition up to 3% by weight of binder further enhanced the mechanical strengths and modulus of elasticity of the concrete. This is due to enhanced pozzolanic reactions between quartz from NS with Portlandites from CCR generating excess calcium silicate hydrates responsible for strength development. However, addition of higher NS above 3% resulted in a decrease in the mechanical properties of the concrete, due to agglomeration and reduced consistency;
(4) Both CCR and NS decrease the porosity of the concrete, resulting in a decrease in water absorption. However, this improvement was for the use of up to 22.5% CCR and up to 3% NS;
(5) The microstructural morphology showed that the combination of NS and CCR in concrete densified its microstructure, refined its pores, and produced more hydration products such as C-S-H;
(6) The optimum mix was obtained using a combination of 15% CCR with 2% NS, which gave the best performance in terms of mechanical strengths, elastic modulus, water absorption, and microstructural refinement;
(7) The use of CCR as cement replacement in concrete reduces the cost and CO_2_ emission of the concrete. NS on the other hand increases the concrete’s cost but does not have much effect on the CO_2_ emission;
(8) The developed concrete can be used for structural applications with a strength of more than 30 MPa where up to 30% cement can be replaced with CCR with the addition of NS.

## 5. Limitations of the Study

This study was limited to normal strength concrete with a target strength of 30 MPa at 28 days. The CCR was obtained from only one source, and likewise the NS. The maximum amount of CCR was limited to 30% as a partial replacement by weight of cement, and the NS was limited to 4% as additive by weight of cementitious materials. The research was also limited to investigating the effect of the hybrid of CCR and NS on the mechanical strength, water absorption, MoE, and microstructural evaluations. The developed concrete can be used for structural applications where up to 22.5% CCR can replace cement with up to 3% NS added. The developed concrete is limited to use for structural applications, with concrete design strength not exceeding 30 MPa at 28 days.

## Figures and Tables

**Table 1:** Chemical properties of materials.

Oxide	Chemical Compositions (%)		
	Cement	CCR	NS
SiO_2_	20.76	3.66	99.8
Al_2_O_3_	5.54	2.56	0.04
Fe_2_O_3_	3.35	1.54	0.005
CaO	61.4	89.13	-
MgO	2.46	-	-
K_2_O	0.76	0.22	-
Na_2_O	0.19	-	-
SO_3_	-	0.54	-
TiO_2_	-	-	-
BaO	-	0.11	-
Loss of Ignition	2.24	2.24	-
Specific Gravity	3.15	2.35	2.28
Specific Surface Area (m^2^/g)	325	290	100 ± 25

**Table 2:** Properties of Aggregate.

Property	Coarse Aggregate	Fine Aggregate
Maximum Aggregate Size (mm)	19	4.75
Specific Gravity	2.67	2.63
Water Absorption (%)	0.83	1.96
Bulk Density (kg/m^3^)	1450	1560
Fineness Modulus	-	2.32
Mud Content (%)	-	1.1

**Table 3:** Mix Proportioning.

Mix	Variables		Quantities of Materials for 1 kg/m^3^ (kg/m^3^)						
	CCR (%)	NS (%)	Cement (kg/m^3^)	CCR (kg/m^3^)	NS (kg/m^3^)	Fine Aggregate (kg/m^3^)	Coarse Aggregate (kg/m^3^)	Water (kg/m^3^)	SP (kg/m^3^)
M0C0N	0	0	388	0	0	665	1258	178	5.82
M0C1N	0	1	388	0	3.88	665	1258	178	5.88
M0C2N	0	2	388	0	7.76	665	1258	178	5.94
M0C3N	0	3	388	0	11.64	665	1258	178	5.99
M0C4N	0	4	388	0	15.52	665	1258	178	6.05
M7.5C0N	7.5	0	388	29.1	0	665	1258	178	6.26
M7.5C1N	7.5	1	358.9	29.1	3.88	665	1258	178	5.88
M7.5C2N	7.5	2	358.9	29.1	7.76	665	1258	178	5.94
M7.5C3N	7.5	3	358.9	29.1	11.64	665	1258	178	5.99
M7.5C4N	7.5	4	358.9	29.1	15.52	665	1258	178	6.05
M15C0N	15	0	358.9	58.2	0	665	1258	178	6.26
M15C1N	15	1	329.8	58.2	3.88	665	1258	178	5.88
M15C2N	15	2	329.8	58.2	7.76	665	1258	178	5.94
M15C3N	15	3	329.8	58.2	11.64	665	1258	178	5.99
M15C4N	15	4	329.8	58.2	15.52	665	1258	178	6.05
M22.5C0N	22.5	0	329.8	87.3	0	665	1258	178	6.26
M22.5C1N	22.5	1	300.7	87.3	3.88	665	1258	178	5.88
M22.5C2N	22.5	2	300.7	87.3	7.76	665	1258	178	5.94
M22.5C3N	22.5	3	300.7	87.3	11.64	665	1258	178	5.99
M22.5C4N	22.5	4	300.7	87.3	15.52	665	1258	178	6.05
M30C0N	30	0	300.7	116.4	0	665	1258	178	6.26
M30C1N	30	1	271.6	116.4	3.88	665	1258	178	5.88
M30C2N	30	2	271.6	116.4	7.76	665	1258	178	5.94
M30C3N	30	3	271.6	116.4	11.64	665	1258	178	5.99
M30C4N	30	4	271.6	116.4	15.52	665	1258	178	6.05

**Table 4:** Cost and CO_2_ Emissions of Constituent Materials.

Materials	Cement	CCR	NS	Fine Aggregate	Coarse Aggregate	Water	SP
Cost $/kg	0.099	0	1.0	0.03	0.04	-	1.2
CO_2_ emission (kg CO_2_/kg)	1 [64,65,66]		0.00084 [18,67]	0.0139 [18,68]	0.0408 [18,68]	0.000196 [18,68]	-

**Table 5:** Cost and CO_2_ Emissions for CCR NS Concrete Mixes.

Mix	Cost ($/m^3^)	CO_2_ Emission (kg/m^3^)
M0C0N	113.3	448.605
M0C1N	117.3	448.608
M0C2N	121.2	448.611
M0C3N	125.1	448.615
M0C4N	129.0	448.618
M7.5C0N	113.7	450.496
M7.5C1N	114.4	421.400
M7.5C2N	118.3	421.403
M7.5C3N	122.2	421.406
M7.5C4N	126.2	421.409
M15C0N	110.8	423.288
M15C1N	111.5	394.191
M15C2N	115.4	394.194
M15C3N	119.4	394.198
M15C4N	123.3	394.201
M22.5C0N	107.9	396.079
M22.5C1N	108.6	366.983
M22.5C2N	112.6	366.986
M22.5C3N	116.5	366.989
M22.5C4N	120.4	366.992
M30C0N	105.0	368.871
M30C1N	105.7	339.774
M30C2N	109.7	339.777
M30C3N	113.6	339.781
M30C4N	117.5	339.784
